# Advances in Gene Delivery Methods to Label and Modulate Activity of Upper Motor Neurons: Implications for Amyotrophic Lateral Sclerosis

**DOI:** 10.3390/brainsci11091112

**Published:** 2021-08-24

**Authors:** Mouna Haidar, Aida Viden, Bradley J. Turner

**Affiliations:** 1 Florey Institute of Neuroscience and Mental Health, University of Melbourne, Parkville, VIC 3052, Australia; mouna.haidar@florey.edu.au (M.H.); aida.viden@florey.edu.au (A.V.); 2 Perron Institute for Neurological and Translational Science, Queen Elizabeth Medical Centre, Nedlands, WA 6150, Australia

**Keywords:** amyotrophic lateral sclerosis, motor cortex, upper motor neurons, retrograde tracing, viral vectors, reporter mouse models

## Abstract

The selective degeneration of both upper motor neurons (UMNs) and lower motor neurons (LMNs) is the pathological hallmark of amyotrophic lateral sclerosis (ALS). Unlike the simple organisation of LMNs in the brainstem and spinal cord, UMNs are embedded in the complex cytoarchitecture of the primary motor cortex, which complicates their identification. UMNs therefore remain a challenging neuronal population to study in ALS research, particularly in the early pre-symptomatic stages of animal models. A better understanding of the mechanisms that lead to selective UMN degeneration requires unequivocal visualization and cellular identification of vulnerable UMNs within the heterogeneous cortical neuronal population and circuitry. Here, we review recent novel gene delivery methods developed to cellularly identify vulnerable UMNs and modulate their activity in various mouse models. A critical overview of retrograde tracers, viral vectors encoding reporter genes and transgenic reporter mice used to visualize UMNs in mouse models of ALS is provided. Functional targeting of UMNs in vivo with the advent of optogenetic and chemogenetic technology is also discussed. These exciting gene delivery techniques will facilitate improved anatomical mapping, cell-specific gene expression profiling and targeted manipulation of UMN activity in mice. These advancements in the field pave the way for future work to uncover the precise role of UMNs in ALS and improve future therapeutic targeting of UMNs.

## 1. Introduction

The initiation and control of voluntary movement is regulated by large pyramidal cells (or Betz cells in humans) residing in Layer 5 of the primary motor cortex, also known as upper motor neurons (UMNs), via their monosynaptic projections toward spinal cord targets along the corticospinal tract (CST). This descending neuromotor pathway is tightly regulated by UMNs and is integral for healthy motor functioning in humans. Indeed, the early and progressive degeneration of UMNs is a pathological hallmark of the fatal neurodegenerative disorder amyotrophic lateral sclerosis (ALS) [1,2,3,4,5]. The initial degeneration of UMNs has been linked to the subsequent degeneration of spinal motor neurons, or lower motor neurons (LMNs), via trans-synaptic glutamate excitoxicity, representing the central premise for the ‘dying-forward’ hypothesis in ALS [6].

LMNs are anatomically well-organised within the simple cytoarchitecture of the brainstem and spinal cord, although their neurophysiology remains complex. Their cell bodies are located either in the cranial nuclei (mainly III, IV, V, VI, VII, XII) or in the ventral horns of the brainstem and spinal cord, respectively. LMNs can be readily identified based on their anatomical, morphological and physiological characteristics, as well as their expression of selective markers such as choline acetyltransferase (ChAT, [7]) and transcription factor homeobox gene HB9 [8], whereas UMNs residing within the complex and complicated organisation of the cortical circuitry lack selective or specific cell markers and are virtually morphologically indistinguishable from other pyramidal neurons. This inability to identify UMNs has proved challenging to precisely define vulnerable cortical neurons and investigate their role in ALS. If we can precisely identify and target vulnerable UMNs under healthy and diseased states, such as ALS, we can better unravel the precise role of UMNs in ALS and improve future therapeutic applications targeting vulnerable UMNs.

The ability to label and manipulate neuronal circuits within the central nervous system (CNS) has come of age with the advancement of various technologies, including the delivery of viral vectors to discrete neural pathways, and the use of transgenic reporter mouse models. These tools are important for dissecting the specific function of UMNs both in physiological conditions and disease. Here, we will review exciting advances in genetic labelling techniques to help unambiguously visualize and identify UMNs within the complex structure of the cerebral cortex (summarized in Table 1). We also discuss how gene delivery advances in conjunction with various genetic modifying technologies allow for the specific modulation of UMN activity and their critical implications for ALS.

## 2. Retrograde Tracers

After the first discovery of retrograde tracing approaches and molecular dyes [36], retrograde tracing began to reveal the extent of the activity of neurons within their neural network and circuitry, highlighting one of the main advantages of these techniques. Retrograde tracing relies on the principle of retrograde axonal transport and can be used to “backfill” the soma of neurons within complex neural circuits by targeting their axonal projections. Commonly used retrograde tracing techniques include bacterial toxin fragments (e.g., cholera toxin subunit B, CTB and tetanus toxin), fluorescent retrograde tracers (e.g., fluorogold, FG and CTB-conjugated with fluorophores) and micro- and nano- particles (e.g., microbeads).

These approaches can be used to selectively identify UMNs by targeting their axonal projections within the CST. Indeed, the origins of the pyramidal tract and the detailed mapping of the CST was first described in early studies with the use of retrograde tracing techniques [37], reviewed in detail previously in [38]. The diversity of Layer 5 projection neurons has been closely mapped using CTB fluorophore conjugates and fluoro-Ruby, and coupled with electrophysiology to identify unique intrinsic properties of retrogradely labelled UMNs making corticospinal projections, compared to cortico-thalamic, -striatal and local cortico-cortical projections [9]. Using FG retrograde labelling, Zang and Cheema reported the first evidence for UMN degeneration in transgenic mutant superoxide dismutase 1 (SOD1^G93A^) mice [12]. A significant and progressive loss of corticospinal neurons was detected as early as postnatal day 60 (P60) in SOD1^G93A^ mice. Furthermore, loss of bulbospinal pathways was also reported from P60 in SOD1^G93A^ mice [12], suggesting that both corticospinal and bulbospinal systems degenerate early in this mouse model of ALS. Ozdinler and colleagues also labelled the somata of UMNs and their proximal apical dendrites to identify vulnerable UMNs in presymptomatic SOD1^G93A^ mice [11]. More recently, Jara and colleagues used retrograde labelling by injection of fluorescent microspheres targeted to the CST to specifically label UMNs in presymptomatic SOD1^G93A^ mice [13]. They coupled retrograde labelling with laser scanning photostimulation, electrophysiology and microarray gene expression profiles on fluorescence activated cell sorted (FACS) purified UMNs to assess extrinsic and intrinsic changes to UMN function in SOD1^G93A^ mice, respectively. In a separate study, FACS-isolated UMNs labelled with fluorescent microbeads in SOD1^G86R^ mutant mice revealed significant RNA metabolism and novel splicing alterations [14]. By combining retrograde labelling with gene expression profiling techniques, these pioneering studies are beginning to define molecular factors and unique markers of vulnerable UMNs and at different stages of the disease in mouse models of ALS. Lastly, identification of UMNs in SOD1^G86R^ mice using FG retrograde tracing along with comprehensive histology and electrophysiology to concurrently assess LMN degeneration revealed that UMN and LMN degeneration may be somatotopically related and that UMN loss likely precedes LMN degeneration [15].

Collectively, these studies highlight the strength of retrograde tracing to selectively identify UMNs based on their target projections within the complex descending neuromotor circuit. They also demonstrate the innovative and creative ways in which retrograde tracing techniques to visualize and label UMNs can be combined with diverse techniques to record their physiological characteristics or gene expression profiles. While UMNs can be anatomically identified based on the uptake of various retrograde tracers to backfill the somata of UMNs, this approach is technically laborious, leads to variable UMN labelling efficiency and may be confounded by axonal pathology and transport defects occurring in ALS. The use of viral vectors for gene delivery can overcome some of these limitations.

## 3. Viral Vectors for Gene Delivery

Recombinant adeno-associated virus (AAV) technology is a powerful tool that can be used to target and manipulate specific neuron subtypes as defined by gene expression, anatomical location and connectivity. The ability to achieve specificity of targeted neuronal populations depends on multiple factors, including (i) spatial targeting of AAV delivery typically via stereotaxic surgery, (ii) dose and titration, (iii) AAV serotype (iv) promoters driving gene expression, and (v) AAV-mediated retrograde transduction [39]. Early comparative studies have assessed various AAV serotypes and neuron-specific promoters [40,41,42], including calcium/calmodulin-dependent protein kinase II subunit alpha (CAMKIIα) and human synapsin (hSyn) [16], for the efficient targeting of the cerebral cortex in mice and primates. In a comparative study of seven AAV serotypes, AAV1 was identified as the optimal serotype for transducing cortical neurons and UMNs with green fluorescent protein (GFP) expression detectable in CST fibres within the cervical spinal cord [17]. In a study screening AAV2 serotypes for UMN labelling, it was demonstrated that AAV2-2 most efficiently transduces corticospinal neurons in mice with enhanced GFP (eGFP) driven by the chicken β-actin (CBA) promoter [18]. More recently, a side-by-side comparison of four commonly used ubiquitously expressed neuronal promoters packaged in AAV1 were assessed for their transduction efficiency of UMNs in mice, involving hSyn, human cytomegalovirus (hCMV), short CMV early enhancer/chicken β-actin (sCAG) and murine phosphoglycerate kinase (mPGK) [19]. The hSyn promoter was most selective for UMNs. While ubiquitously expressed promoters are widely used and accepted to target cortical neurons, they target both inhibitory and excitatory neurons [43,44] and therefore lack the specificity for targeting UMNs which are solely excitatory.

**Figure 1:**
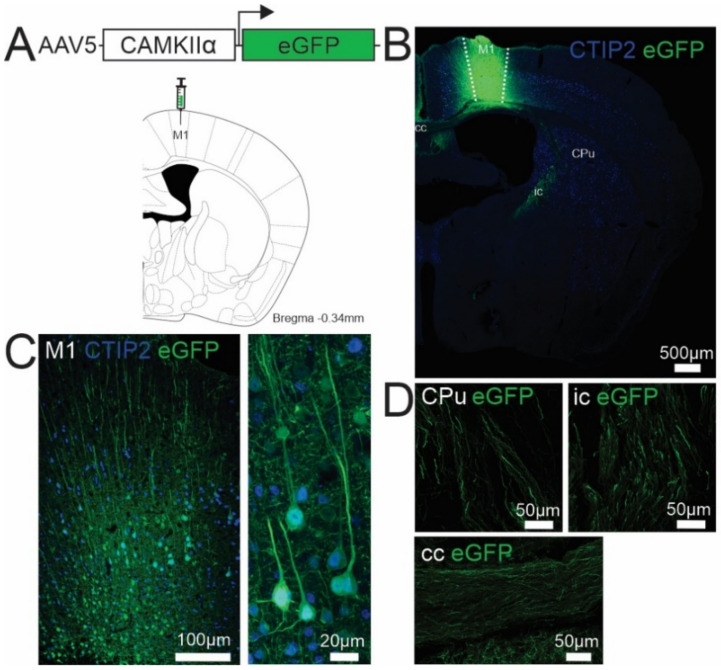
(**A**) Schematic representation of AAV5-CAMKIIα-eGFP viral construct and coronal section depicting stereotaxic targeting to the mouse primary motor cortex (M1, coronal brain image adapted from the mouse brain atlas, Paxinos and Franklin 2001). (**B**,**C**) Representative immunofluorescence images showing eGFP (green) expression is anatomically confined to the M1 and within the somata and atypical dendrites of CTIP2 (blue, a marker for Layer 5 cortical neurons)-positive Layer 5 neurons. (**D**) eGFP-positive fibres are expressed within the caudate putamen (CPu), internal capsule (ic) and corpus callosum (cc).

The use of AAVs driven by the well validated glutamate/excitatory specific neuronal CAMKIIα promoter overcomes this limitation [45]. For example, in our studies using an AAV5 with eGFP under the control of CAMKIIα promoter and stereotaxically targeted to the mouse primary motor cortex (Figure 1A), this allowed for targeting and visualization of the soma and proximal apical dendrite of large pyramidal Layer 5 neurons within the primary motor cortex (Figure 1B,C), and their defined CST axonal projections within the internal capsule (ic), corticostriatal projections within the caudate putamen (CPu), and corticocortical projections within the corpus callosum (cc) (Figure 1D).

In addition to allowing for the specific targeting of neuron subtypes based on gene expression and location, AAV technology can be easily coupled with gene modifying technologies to modulate the activity of genetically identified neuronal populations and circuits. For example, a powerful test to determine the specific effects of UMNs on motor behaviour can be assessed by the acute or chronic activation or inactivation of genetically defined UMNs. This can be achieved by coupling AAV-mediated targeting of UMNs with chemogenetics or designer receptors activated by designer drugs (DREADDs) technology [46] to modulate the activity of UMNs and their circuitry with spatial and temporal control in vivo. Namely, selectively enhancing or inhibiting the activity of UMNs by G-protein-coupled receptors (GPCRs) by inert compounds can be used to modulate neuronal excitability and potentially downstream excitotoxicity occurring in ALS.

An AAV approach optimized based on serotype and a glutamate-specific neuronal promoter, as well as spatial targeting as described above, targets UMNs within a heterogeneous pyramidal/glutamatergic cortical population and therefore lacks specificity. For example, whilst an AAV5-CAMKII-eGFP approach allows for the specific targeting of excitatory projection neurons within the primary motor cortex, it does not differentiate between the corticospinal, -striatal and -cortical tracts originating from Layer 5 projection neurons (Figure 1D). This limitation can be overcome with the use of viral retrograde transduction by allowing projection-specific targeting of UMNs. For example, various AAV-mediated retrograde tracers have been developed and have shown specificity to UMNs when targeted to the dorsal CST [38,47]. A combinatorial viral approach can be used to manipulate the activity of retrogradely targeted UMNs. This approach consists of a viral mediated retrograde tracing approach and DREADD technology via Flp- or Cre- mediated recombination. For example, a canine adenovirus type 2 expressing Cre recombinase (CAV2-Cre) can be injected into the projection region along the pyramidal tract, followed by the injection of a locally transducing Cre-dependent viral vector expressing DREADDs in the primary motor cortex. This results in specific targeting of Layer 5 pyramidal neurons projecting to the spinal cord via the CST and characteristic of all UMNs (shown previously be us in [5]) and upon inert ligand activation, can be modulated in vivo. Retrograde approaches can also be integrated with optogenetics for selective acute inhibition or excitation of projections between brain regions or cells defined by projecting from one brain region to another [48]. For example, to assess the therapeutic effect of optogenetically activating the intact CST after a stroke in adult rats, selective expression of the light-gated channel that can depolarize neurons, channelrhodopsin-2 (ChR2), in corticospinal-projecting neurons in rats was achieved by a dual viral approach, whereby a retrograde AAV9-CAMKIIα-Cre vector was injected in the cervical spinal cord, followed by the injection of a Cre-recombinase dependent ChR2 vector in the ipsilateral motor cortex [20]. This approach could be applied to ALS mouse models to help elucidate the effects of inhibiting or exciting UMNs defined by their projections along the CST, respectively. Additionally, a retrograde CAV2-Cre approach has also been used in combination with Cre-dependent genetically encoded calcium indicator (GCaMP6) construct to visualize and record the activity of UMNs in SOD1^G93A^ mutant mice using two-photon calcium imaging [10]. Despite the significant contributions viral vector technologies continue to make to the neuroscience field, AAV approaches have inherited experimental limitations, such as between-subject and -experimenter variability, variability of somatic transgenesis, the invasiveness of surgical injection of the AAV into the tissue of interest, potential toxicity arising from AAV-mediated gene overexpression, potential immunogenicity of AAV capsid, and the inability to transduce neurons spanning a large anatomical region. These limitations can be overcome with the use of germ-line transgenic mouse lines with promoter driven transgenes.

## 4. Transgenic Reporter Mice

An important advance in genetic labelling has been the introduction of transgenic reporter mouse lines in which a fluorescent protein is constitutively and stably expressed in targeted neuron subpopulations in the CNS. Importantly, compared to AAV approaches which rely on accurate surgical targeting, genetic labelling approaches ensure the reproducibility of findings, highlighting their major advantage. Transgenic reporter mice expressing spectral variants of GFP (including yellow fluorescent protein, YFP) under the control of the Thy-1 promoter [21] have been widely used to identify and characterise cortical neurons in early studies [22,23,24]. Importantly, Thy1-YFP mice have been extensively used to study the motor system in ALS mouse models. Thy1-YFP mice crossbred with SOD1^G93A^ mice were used to label motor axons [25,26], UMNs and CST axons [11]. Furthermore, Thy1-YFP mice crossbred with mutant TAR DNA binding protein 43 (TDP-43^A315T^) mice allowed the detection of early UMN pathology where spine loss preceded somata degeneration, implicating synapse pathology early in this mouse model of ALS [27]. Whilst Thy1-YFP mice have been a useful tool for studying motor systems, the Thy1 promoter labels a heterogeneous population of cortical neurons and their axons along the descending motor axis [23,24], and therefore fails to specifically identify vulnerable UMNs in ALS. In addition, Thy1-YFP expression per se leads to spontaneous neurodegeneration and an altered response to axonal injury in some lines, which may confound experiments using neurological disease models.

To overcome this important limitation, Yasvoina and colleagues generated and characterised ubiquitin carboxy-terminal hydrolase L1 (UCHL1) mice which express enhanced GFP (eGFP) under the murine UCHL1 promoter [28]. UCHL1 was chosen based on its relatively restricted expression to large Layer 5 pyramidal neurons, characteristic of all UMNs [49]. Using retrograde labelling studies, UCHL1-eGFP expression was confined to UMNs projecting along the CST, and eGFP positive neurons expressed markers specific to UMNs. Importantly, UCHL1-eGFP mice crossbred with SOD1^G93A^ mice demonstrate eGFP positive neurons are vulnerable in ALS disease progression [28]. Since their initial characterization, UCHL1-eGFP mice have been extensively used to study UMN degeneration in animal models of ALS, including alsin knockout (KO) and transgenic TDP-43^A315T^ mice [13,29,30,31,50], and therefore are an invaluable tool for uncovering the precise role of UMNs in ALS. While UCHL1-eGFP mice provide an appropriate model to label UMNs, eGFP and therefore UCHL1 are also expressed in neurons of the somatosensory cortex, striatum and spinal cord [28], which limits their utility to anatomical mapping studies, rather than functional studies.

Another reporter line used to identify Layer 5 projection neurons including UMNs is the transcription factor, with forebrain embryonic zinc finger protein (Fezf2) mice expressing GFP under the Fezf2 promoter. Fezf2 is highly expressed predominately in Layer 5, including pyramidal tract projection neurons in Layer 5B, which UMNs occupy, as well as intratelencephalic projection neurons in Layer 5A, in addition to Layer 6 neurons [32,33,51,52]. Genetic ablation of Fezf2 resulting in the complete absence of Layer 5 projection neurons in an ALS mouse models achieved by crossbreeding Fezf2 KO mice with SOD1^G93A^ mice paradoxically delayed disease onset and progression [53], further highlighting the specificity of Fezf2 to identify vulnerable UMNs within the diverse architecture of Layer 5 in ALS.

Thirdly, mu-crystallin (Crym)-GFP reporter mice show specifically labelling of pyramidal neurons in Layer 5 of the cerebral cortex, including the primary motor cortex, and within the CST, evidenced by axonal expression in the dorsal, lateral and ventral funiculi [34]. This expression profile was mirrored by Crym-CreER^T2^; TdTomato mice in which pyramidal neurons were labelled in Layers 5 and 6 throughout the cortex [35]. Furthermore, excision of the mutant SOD1^G37R^ transgene specifically from corticofugal projection neurons including UMNs was achieved by cross breeding these Crym-CreER^T2^ mice with Cre-dependent floxed SOD1^G37R^ mice [35]. Although mutant SOD1 silencing in Crym-positive UMNs did not affect disease onset or outcome, in contrast to LMN suppression, this study highlights the usefulness of Crym as a potential UMN selective marker for ALS research.

## 5. Functional Targeting of UMNs

To modulate the activity of genetically labelled neurons, UMN-specific reporter mice driving Cre recombinase can be crossbred with Cre-dependent human muscarinic receptor or light-sensitive rhodopsinmouse lines. Recently, studies using SOD1^G93A^ mice crossbred with ChAT-Cre mice and which received an AAV Cre-dependent excitatory DREADD intraspinal injection helped to elucidate the neuroprotective properties to LMNs in ALS following synaptic restoration [54]. Such an approach can be applied to the ALS field to better elucidate the neuroprotective or adverse effects of modulating UMN activity. Indeed, Cre reporter mice, including mice driving Cre under the control of a CAMKIIα promoter (CAMKIIα-Cre), were crossbred with Cre-dependent hM3Dq-mCherry mice to localise hM3Dq expression to the soma and dendrites within cortical and hippocampal neurons and identified by the presence of mCherry [55], demonstrating the feasibility of this approach to modulate the activity of UMNs. Similarly, crossbreeding of CAMKIIα- or EMX1-Cre mice with mice with hM3Dq or hM4Di under the control of the ubiquitous promoter CAG with a loxP site-flanked stop signal upstream of a HA-tagged DREADD and a fluorescent promoter, mCitrine (CAG-LSL-HA-DREADD-mCitrine), results in the removal of the stop sequence and therefore expression of hM3Dq or hM4Di restricted to cortical and hippocampal neurons or excitatory cortical projection neurons, respectively [56]. In addition, to assess the role of LMN firing during locomotor activity in neonatal mice, mice expressing a floxed light-gated proton or chloride (archaerhodopsin or halorhodopsin, respectively) or channelrhodopsin were crossed with ChAT-Cre or Islet1 transcription factor Isl1-Cre mice to hyperpolarize or depolarize LMNs, respectively [57]. Consequently, the use of conditional genetics relying on Cre-dependent recombination with UMN specific reporter lines to selectively and reliably target UMNs will achieve widespread targeting of UMNs and overcome some of the inherited limitations of AAV-driven methods. Such work has the potential to yield tremendous insights into the precise role of UMNs in ALS pathogenesis and to provide novel therapeutic targets.

## 6. Conclusions

UMN degeneration is an important pathophysiogical and diagnostic biomarker of sporadic and familial ALS. Studying UMN pathology in the presymptomatic and early phases of ALS, however, has proved to be challenging. Unlike LMNs, UMNs are difficult to identify amongst the heterogeneous and complex nature of the cerebral cortex and their downstream circuits. This has greatly hindered the ability to uncover the precise role of UMNs in ALS pathogenesis and has therefore stalled the overall advancement in treatments targeted to vulnerable UMNs.

Tremendous progress has been made towards the goal of unambiguously cellularly identifying UMNs. Here, we reviewed exciting progress in the field to genetically label and manipulate the activity of UMNs in relevant in vivo ALS models. We described advancements in the use of retrograde labelling, viral vectors for gene delivery and transgenic reporter mouse lines and the ability to couple these techniques with gene-modifying technologies to allow for the specific and targeted manipulation of UMNs activity. Importantly, we highlight the strengths and limitations of these various technologies to be taken into consideration when implementing them in experiments and interpreting data.

The main challenge in the field remains the identification of cell-type-specific mechanisms and unique cellular adaptations underlying vulnerable UMNs in ALS. Recent advances in molecular profiling techniques such as translational ribosome affinity purification (TRAP, [58]), in combination with genetic labelling approaches to target UMNs, have the tremendous potential to elucidate molecular profiles of vulnerable UMNs in preclinical ALS models. This application paves the way to, for the first time, discover new therapeutic compounds to improve the health of vulnerable UMNs in ALS.

## Figures and Tables

**Table 1:** Gene delivery methods to selectively label and visualize UMNs in vivo.

Retrograde Tracers					
Tracer		Injection Site		Model	Ref.
Cholera toxin subunit B		C3/C4 or T13/L1 C5/C6		Swiss Webster mouse, WT SOD1^G93A^ mouse	[9] [10]
Fluoro-Ruby		C3/C4 or T13/L1		Swiss Webster mouse, WT	[9]
Fluorescent microsphere, (LumaFluor)		C4/C6 [11] or C5/C6 [10]		SOD1^G93A^ mouse	[10,11]
Fluoro-Gold		T12 SC [12] or C3/C4 [13] C3/C4 or L1/L2		SOD1^G93A^ mouse SOD1^G86R^ mouse	[12,13] [14,15]
**Viral vectors**					
**Serotype**	**Promoter**	**Reporter gene**	**Injection site**	**Model**	**Ref.**
AAV1 or 9 AAV1 or 9	CAMKII * hSyn *	hrGFP hrGFP	M1	Marmoset, C57BL/6J mouse, macaque	[16]
AAV1	CMV	eGFP	M1	Sprague Dawley rat	[17]
AAV2-2	CBA	eGFP	M1	SOD1^G93A^ mouse	[18]
AAV1	hSyn *	eGFP	M1	Lister Hooded rat and C57BL/6J mice	[19]
AAV9 (retrograde)	CAMKIIα	Cre	cervical SC	Long Evans rat	[20]
CAV2 (retrograde)	-	Cre	C5/C6	SOD1^G93A^ mouse	[10]
**Transgenic Reporter Mice**					
**Locus**	**Reporter Gene**		**Model**		**Ref.**
Thy1	YFP		Thy1-YFP		[21,22,23,24] #
			Thy1-YFP; SOD1^G93A^		[25,26]
			Thy1-YFP; TDP-43^A315T^		[27]
UCHL1	eGFP		UCHL1-eGFP; SOD1^G93A^		[13,28,29]
			UCHL1-eGFP; alsin KO		[30]
			UCHL1-eGFP; TDP-43^A315T^		[29,31]
Fezf2	GFP		Fezf2-GFP		[32,33]
Crym	GFP CreER^T2^		Crym-GFP Crym-CreER^T2^; TdTomato Crym-CreER^T2^; SOD1^G37R^		[34] [35] [35]

* Comparative studies; serotype and promoter most selective for UMNs listed. # Various spectral variants including the Thy1-RFP, -GFP, and -CFP phenotypes. Cortical expression in Thy1-YFP mice is largely restricted to neurons in Layer 5 and listed here.

## Data Availability

Unavailability Statement.
